# Supplementary material for: Key Factor Study for Generic Long-Acting PLGA Microspheres Based on a Reverse Engineering of Vivitrol®
Source: Molecules. 2021 Feb 25;26(5):1247. doi: 10.3390/molecules26051247 (PMC7975983; doi:10.3390/molecules26051247)
Supplement: Supplementary file 1 [file molecules-26-01247-s001.pdf]

*Supplementary*

# Key factor study for generic long-acting PLGA microspheres based on a reverse engineering of Vivitrol®

Yabing Hua[1], Zengming Wang[1], Dan Wang[1], Xiaoming Lin[1], Boshi, Liu[1], Hui Zhang[1], Jing Gao[1*], Aiping Zheng[1*]

[1] State Key Laboratory of Toxicology and Medical Countermeasures, Beijing Institute of Pharmacology and Toxicology, Beijing, China, 27th Taiping Road, Haidian District, Beijing, 100850, China

* Corresponding author: Aiping Zheng

Postal Address: 27th Taiping Road, Haidian District, Beijing 100850, China

E-mail: apzheng@163.com

Office Phone: +86 10 66931694. Mobile: +86 13520467936

*Corresponding author: Jing Gao

Postal Address: 27th Taiping Road, Haidian District, Beijing 100850, China

E-mail: gjsmmu@126.com

Office Phone: +86 10 66874665. Mobile: +86 13817029922

Verification of HPLC methodology

Table S1 Verification of HPLC method to determine naltrexone*

| | | Results |
|---|---|---|
| System applicability | | After 6 injections of the reference solution, the RSD of retention time and peak area is less than 1%, and the trailing factor is between 0.9 and 1.1. In addition, the separation degree is more than 4. |
| Linearity and range | | When naltrexone is in the range of 1-100 μg/mL, the linear equation is y = 35.772x + 43.091, and the linear regression coefficient r=0.9998, indicating a good linear relationship between concentration and peak area. The limit of detection is 100 ng/mL and the limit of quantitation is 1 μg/mL. |
| Precision | Repeatability | The RSD of 6 samples of solution was 0.27% (< 1.0%). |
| | Intermediate precision | Another experimenter measured the samples with different instruments on other dates, and the RSD of six samples was 0.43% (< 2.0%). |
| Recovery rate | | Both of the intra-group and inter-group recovery rate are between 98% and 102%, and the RSD of them is 0.49% (< 2.0%). |

| | |
|---|---|
| Specificity | The blank solvent and excipient solution did not interfere with the determination of naltrexone, and the resolution between the main peak and the adjacent peak was 5 (> 1.5). |

*The verification results of HPLC methodology meet the requirements of ICH Q2.

Verification of Microsphere determination methodology

Table S2 Particle size and distribution of microspheres determined by different amounts of microspheres

| | 50 mg | 100 mg | 150 mg | 200 mg | 300 mg |
|---|---|---|---|---|---|
| $D_{10}$(μm) | - | - | 19.01 | 19.43 | 19.48 |
| $D_{50}$(μm) | - | - | 35.03 | 35.02 | 35.74 |
| $D_{90}$(μm) | - | - | 62.46 | 60.06 | 62.75 |
| Span | - | - | 1.24 | 1.16 | 1.21 |

When the weight of microsphere is less than 100 mg, the determination condition cannot be reached. When the weight of microsphere is 200 mg, the span value measured is relatively small, and the particle size result is stable. While using a smaller amount of microspheres, it can achieve a certain shading, and will not precipitate and agglomerate. Therefore, the amount of microsphere is determined to be 200 mg.

Table S3 Particle size and distribution of microspheres determined by different suspending agents

| | 0.1 % PVA | Injection diluent | 0.1 % Tween-20 | 0.5 % Tween -20 | 1.0 % Tween -20 |
|---|---|---|---|---|---|
| $D_{10}$(μm) | 20.05 | 18.21 | 20.73 | 19.01 | 19.08 |
| $D_{50}$(μm) | 36.43 | 35.25 | 37.19 | 35.03 | 34.90 |
| $D_{90}$(μm) | 63.06 | 68.58 | 62.71 | 62.46 | 60.09 |
| Span | 1.28 | 1.43 | 1.23 | 1.24 | 1.18 |

When 1.0% Tween-20 is used as suspension agent, the span value is the lowest, indicating that the particle size distribution is good. The selected suspension agent is 1% Tween-20. The suspending agent alone does not affect the determination of this method.

Table S4 Particle size and distribution of microspheres with different stirring speed

| | 1500 rpm | 1800 rpm | 2100 rpm |
|---|---|---|---|
| $D_{10}$(μm) | 19.22 | 20.81 | 19.26 |
| $D_{50}$(μm) | 35.71 | 36.83 | 35.36 |
| $D_{90}$(μm) | 65.46 | 71.55 | 62.21 |
| Span | 1.19 | 1.18 | 1.12 |

When the rotational speed is 2100 rpm, the span value is the smallest, and the agglomeration phenomenon at low rotational speed is avoided.

Table S5 Precision of particle size determination method

| Times | $D_{10}$(μm) | $D_{50}$(μm) | $D_{90}$(μm) | Span |
|---|---|---|---|---|
| 1 | 47.14 | 79.74 | 128.33 | 1.02 |
| 2 | 46.85 | 79.31 | 127.28 | 1.01 |
| 3 | 46.91 | 79.37 | 127.41 | 1.01 |
| 4 | 46.93 | 79.48 | 127.67 | 1.02 |
| 5 | 46.97 | 79.60 | 128.17 | 1.01 |
| 6 | 46.96 | 79.75 | 128.73 | 1.01 |
| RSD/% | 0.09 | 0.17 | 0.52 | 0.51 |

One sample of Vivitrol® was prepared, and the RSD of six measurements were all less than 1.0%, indicating that the precision of this method was good.

Table S6 Repeatability of particle size determination method

| | $D_{10}$(μm) | $D_{50}$(μm) | $D_{90}$(μm) | Span |
|---|---|---|---|---|
| 1 | 47.04 | 77.32 | 124.54 | 1.00 |
| 2 | 47.59 | 77.60 | 126.58 | 1.02 |
| 3 | 46.78 | 77.38 | 125.42 | 1.02 |
| 4 | 46.98 | 78.18 | 126.83 | 1.02 |
| 5 | 47.79 | 77.61 | 125.80 | 1.01 |
| 6 | 46.55 | 78.20 | 126.96 | 1.03 |
| RSD/% | 0.93 | 0.45 | 0.68 | 1.02 |

Six samples of Vivitrol® were prepared in parallel, and each sample was repeatedly determined for 3 times. The RSD values were all less than 2.0%, which indicated that the method had good repeatability.

Molecular weight of GNM with different PLGA

Table S7 Molecular weight by GNM prepared from PLGA with different molecular weight

| | Mw | PD |
|---|---|---|
| GNM-PLGA (8,000 Da) | 47695±273 | 1.52±0.02 |
| PLGA (8,000 Da) | 83416±1557 | 1.73±0.05 |
| GNM-PLGA (140,000 Da) | 81381±2475 | 1.93±0.16 |
| PLGA (140,000 Da) | 140928±774 | 1.83±0.03 |

We prepared the GNM using PLGA which Mw is 63416±1557 Da, but the detected Mw in the resulted microsphere was only 27695±273 Da (Table S9). When the Mw of PLGA used in preparation increased to 140928±774 Da, the detected Mw of PLGA in GNM became similar with that of Vivitrol® as 81381±2475 Da, which meant that 42.25% molecular weight loss happened in the preparation process.

Morphology of microspheres with different solvent

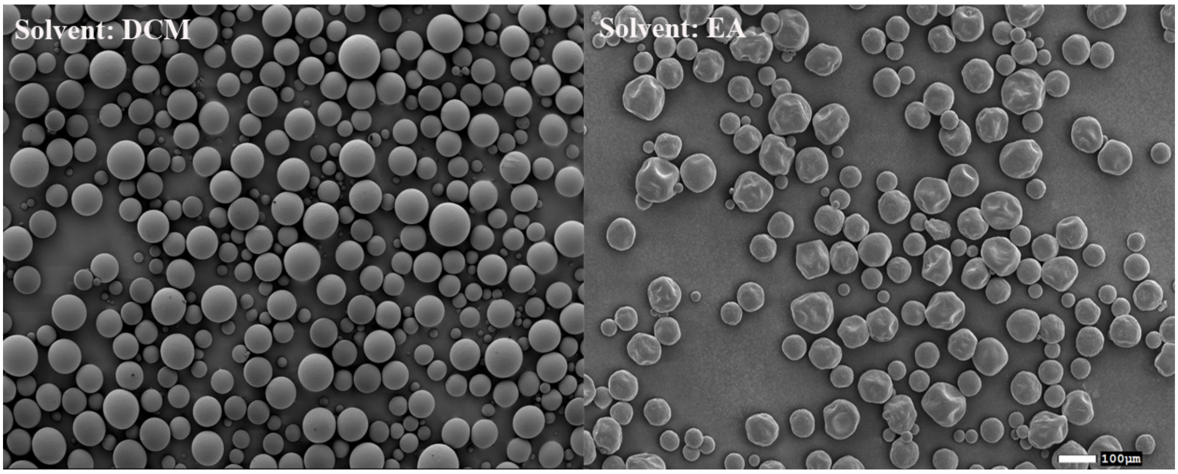


Figure. S1 Morphology of microspheres when the solvent is DCM or EA.
DCM: Dichloromethane, EA: ethyl acetate.

It showed significant difference in morphology with DCM or EA. Microspheres prepared using DCM seemed relatively round, no concavity but porous on the surface, and had apparent difference with that using EA.

Morphology of pressed microsphere before the water contact angle determined

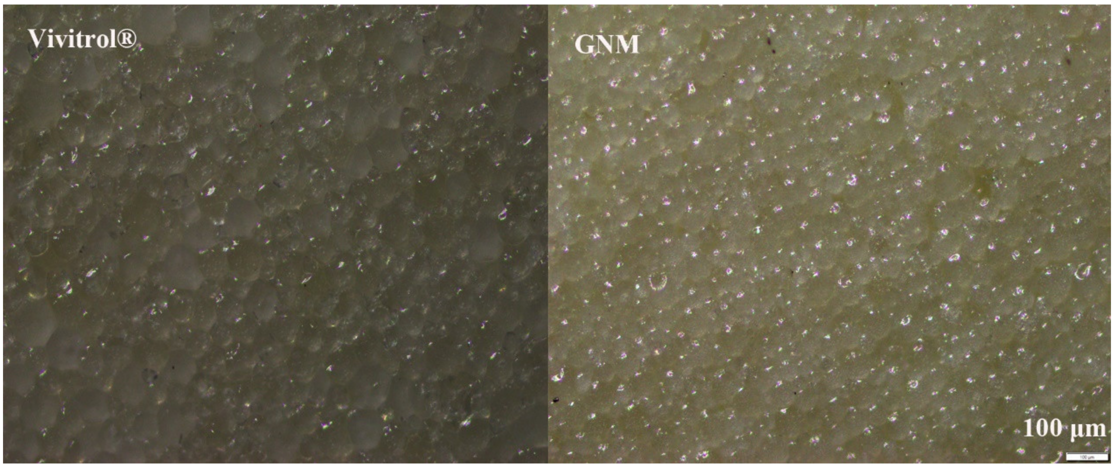


Figure. S2 Morphology of pressed microsphere before the water contact angle determined

Table S8 Contact angle of microspheres before and after drug delivery

| | Contact angle (º) |
|---|---|
| Blank Microspheres | 62.19±0.40 |
| GNM | 55.93±0.38 |
